# LncRNAs in polyploid cotton interspecific hybrids are derived from transposon neofunctionalization

**DOI:** 10.1186/s13059-018-1574-2

**Published:** 2018-11-12

**Authors:** Ting Zhao, Xiaoyuan Tao, Shouli Feng, Luyao Wang, Hui Hong, Wei Ma, Guandong Shang, Shisong Guo, Yuxin He, Baoliang Zhou, Xueying Guan

**Affiliations:** 1 State Key Laboratory of Crop Genetics and Germplasm Enhancement, Cotton Hybrid R & D Engineering Center (the Ministry of Education), College of Agriculture, Nanjing Agricultural University, Nanjing, 210095 Jiangsu China; 2 College of Agriculture and Biotechnology, Zhejiang University, Zhejiang, 210058 Hangzhou China; 3 National Key Laboratory of Plant Molecular Genetics, National Plant Gene Research Center, Institute of Plant Physiology and Ecology, Shanghai Institutes for Biological Sciences, Chinese Academy of Sciences, Shanghai, 200032 China

## Abstract

**Background:**

Interspecific hybridization and whole genome duplication are driving forces of genomic and organism diversification. But the effect of interspecific hybridization and whole genome duplication on the non-coding portion of the genome in particular remains largely unknown. In this study, we examine the profile of long non-coding RNAs (lncRNAs), comparing them with that of coding genes in allotetraploid cotton (*Gossypium hirsutum*), its putative diploid ancestors (*G. arboreum*; *G. raimondii*), and an F_1_ hybrid (*G*. *arboreum* × *G*. *raimondii*, AD).

**Results:**

We find that most lncRNAs (80%) that were allelic expressed in the allotetraploid genome. Moreover, the genome shock of hybridization reprograms the non-coding transcriptome in the F_1_ hybrid. Interestingly, the activated lncRNAs are predominantly transcribed from demethylated TE regions, especially from long interspersed nuclear elements (LINEs). The DNA methylation dynamics in the interspecies hybridization are predominantly associated with the drastic expression variation of lncRNAs. Similar trends of lncRNA bursting are also observed in the progress of polyploidization. Additionally, we find that a representative novel lncRNA XLOC_409583 activated after polyploidization from a LINE in the A subgenome of allotetraploid cotton was involved in control of cotton seedling height.

**Conclusion:**

Our results reveal that the processes of hybridization and polyploidization enable the neofunctionalization of lncRNA transcripts, acting as important sources of increased plasticity for plants.

**Electronic supplementary material:**

The online version of this article (10.1186/s13059-018-1574-2) contains supplementary material, which is available to authorized users.

## Introduction

Interspecific hybridization and polyploidization are known as intrinsic powers behind genome evolution. Polyploidization, also known as whole genome duplication (WGD), is commonly observed in the evolution of angiosperm plants [1]. Polyploidy, especially allopolyploidy, stabilizes the vigor traits created by hybridization. The genomic interactions in hybrids and polyploids trigger a rapid and extensive reprogrammed response, associated with dramatic changes in the epigenetic modifications involved with, but not limited to, the following: DNA methylation, siRNAs, transposable elements (TEs), and histone modification [2, 3]. The integrative results of these genome-wide modifications lead to expression changes in about 20–50% of mRNA, which is the proposed molecular basis for the vigor of hybridization and polyploidization [4, 5].

Small interfering RNAs (siRNAs), especially those derived from TEs, can suppress mobile element activity via RNA-directed DNA methylation (RdDM) [6–8]. In hybrids of *Arabidopsis thaliana*, rice, and maize, expression levels of siRNAs changed dramatically upon polyploidization [9–11]. DNA methylation changes also coincided with activation of TEs in analysis of intraspecific hybrids of *A. thaliana*, rice, and maize [12–16]. However, the impact of hybridization and polyploidization on the lncRNA of whole genomes remains largely unknown.

Long noncoding RNAs (lncRNAs) are typically transcribed from the intergenic regions of the genome, while some lncRNAs originate from the antisense strands of coding genes [17]. In the last few years, lncRNAs have been widely identified in both animal and plant genomes [18–22]. In animal genomes, lncRNAs are associated with X chromosome inactivation [23], disease development [24], etc. Epigenetic modifications on lncRNA are reported to play critical roles on its expression and function. More than 1000 lncRNA genes are found to be hypo-methylated in cancer cell lines recurrently [25]. In plants, lncRNAs are reported to play critical roles in multiple regulation functions, such as developmental regulation [26–28] and both biotic and abiotic stress responses [26, 29–31]. Although the many functions of lncRNA have gradually been elucidated, the exact origin of lncRNA is still obscure.

According to previous genome-wide investigations, lncRNA transcriptomes appeared unique to each species [32, 33]. For example, mRNA similarity between genomes of human and mouse is 92%, but the lncRNA similarity between them is as low as 35% [34]. Less than 6% of zebrafish lincRNAs (long intergenic RNAs) have any detectable sequence similarity to human and mouse lincRNAs [35]. In the comparison of 16 vertebrate species and the sea urchin, > 70% of lincRNAs cannot be traced to homologs in species that diverged > 50 million years ago [36]. Similar trends have been observed in plant species. For example, less than 0.4% of predicted lncRNAs were reported to present in two different tomato species [37]. These data suggest that a genome can generate a large amount of novel lncRNAs efficiently when a new species comes into being. The origin of these species-specific lncRNAs is still unknown.

The latest studies have reported that transposon elements (TEs) might be involved with lncRNA origin and diversification [38–40]. For example, *Xist* originated from a coding gene, *Lnx3*, with accumulated TEs in its exons [41]. We previously found that an NAT (natural antisense transcript) originating from a locus on the coding gene *GhMML3* is associated with TE insertion. This NAT caused the fuzzless seed mutant of *N*_*1*_ by suppressing *GhMML3* expression [42]. TEs are abundant in advanced organisms, especially in plants. For example, TEs comprise 80% of the maize genome and 65% of the cotton genome [43–46]. TEs can be classified as retrotransposons and DNA transposons, each with diverse patterns in sequence and activity [47]. We still do not know which type of TE is related to lncRNA origin.

The behavior of lncRNAs during hybridization and polyploidization provides an important clue to the origin of lncRNA. Here we utilized a simplified model of cotton hybridization and polyploidization to study the origin of lncRNA. Cotton is not only a source of natural and renewable fiber for textiles, but also a fine model for heterosis studies. Regarding evolutionary lineage, upland cotton (*Gossypium hirsutum*, (AADD)_1_, Gh), an allotetraploid species, was formed after the hybridization and polyploidization of its two closest extant progenitors, *G. herbaceum* (A_1_ genome) or *G. arboreum* (A_2_ genome), and *G. raimondii* (D_5_ genome), about 1–1.5 million years ago (MYA) [44, 48]. The two diploid progenitors diverged 6–6.3 MYA [44, 48]. We crossed *G. arboreum* (Ga) accession from Shixiya with *G. raimondii* (Gr), generating an F_1_ hybrid. Using Ga, Gr, (Ga × Gr) F_1_, and Gh (accession Texas Marker-1 (TM-1)), we constructed a system to mimic the evolution of *Gossypium* spp. from diploid to allotetraploid. To determine the origin and behavior of lncRNAs in plant genome evolution, we used methods of interspecific comparative genomics, after identifying 1:1 lncRNA orthologs between species. Based on our integrative analysis of lncRNA sequencing, small RNA sequencing, ChIP-Seq, and DNA methylation data, our results suggested that LINEs arising from TEs play a crucial role in the origin of lncRNAs.

## Results

### The *Gossypium* lineage-specific lncRNA transcriptome

**Fig. 1:**
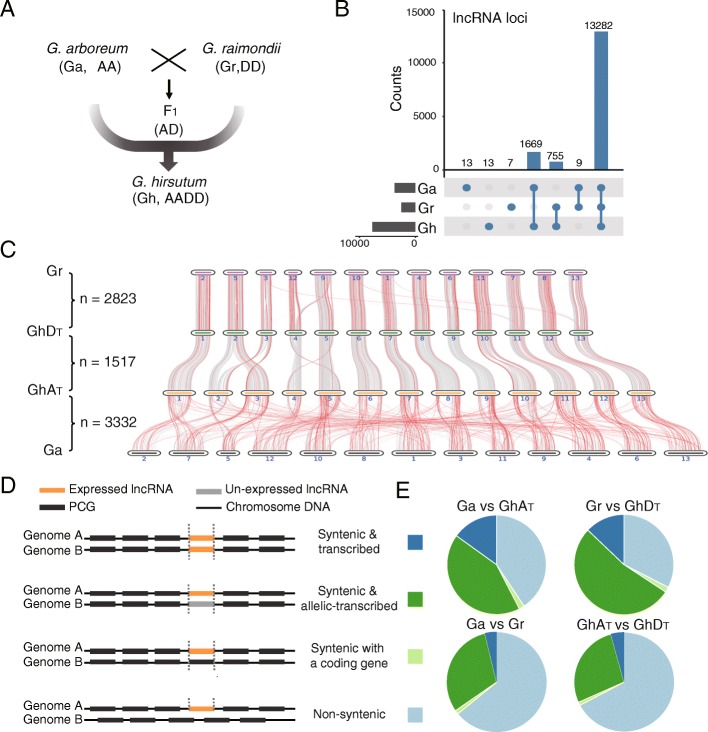
Lineage-specific and allelic-transcribed cotton lncRNA. **a** Schematic model for the diploid species (*Gossypium arboreum*, Ga; *G. raimondii*, Gr; F_1_; and *G. hirsutum*, Gh), F_1_ hybrid, and the allotetraploid species. **b** Histogram showed the lncRNA homologous loci distribution in Ga, Gr, and Gh. *n* represents the number of lncRNA loci. **c** Genome-wide identification of syntenic blocks between Ga, Gr, and Gh. *n* represents syntenic lncRNA in the comparison of different subgenomes. Gray lines represent protein-coding genes (PCGs). Red lines represent lncRNAs. **d** Schematic chart showing the lncRNA homologs classified according to syntenic and expressive pattern. **e** Pie chart showing the number of each classification from panel **e**

We generated ribo-depleted strand-specific RNA-seq libraries of *Gossypium hirsutum*, the ancestors *G. arboreum* and *G. raimondii*, and the F_1_ hybrid using leaf and ovule tissues (Fig. 1a, Additional file 1: Table S1) [49]. Ultimately 8514 lncRNAs in Gh, 4107 in Ga, 2767 in Gr, and 8126 in F_1_ were obtained respectively (Additional file 1: Table S2). Among the predicted lncRNAs, ~ 90% were lincRNAs (Additional file 1: Table S3). The genomic features of these lncRNAs were similar to those identified in previous studies, including low CG content, fewer exons, and lower expression levels compared to protein-coding genes (PCGs) (Additional file 2: Figure S1 A-C) [20, 50]. Approximately 60% of lncRNAs were closed to PCGs (< 5 kb) (Additional file 2: Figure S1D, E). Two thirds of the lncRNAs were overlapped with repetitive TEs (Additional file 2: Figure S1F).

For comparative genome analysis, the genome sequences of *Arabidopsis thaliana*, *Oryza sativa*, and *Theobroma cacao* were selected to test conservation of lncRNA. Less than 3.83% (*n* = 590) of lncRNAs showed homologous sites in *A. thaliana* and *O. sativa*, from which the cotton genome diverged 87 and 115 million years ago (MYA) [51], respectively. But the primary sequences of most lncRNAs (86%, *n* = 13,282) were common to all three cotton genomes (Fig. 1b, Additional file 2: Figure S2A), which diverged ~ 2–8 MYA [44, 48]. A small portion of lncRNAs (12.43%) had homologs in *Theobroma cacao*, in contrast to the much greater number of PCG homologs (69.25% homologs in *T. cacao*) (Additional file 2: Figure S2B). The above data confirmed that cotton lncRNAs are predominantly *Gossypium* lineage-specific.

To obtain a reliable cross-species comparison, the lncRNA loci were classified into syntenic and non-syntenic groups based on their chromosomal locations (Fig. 1c, d, details in method). We first defined the syntenic lincRNA loci, which represent one-to-one homology with lncRNA loci (Fig. 1d, Additional file 1: Table S4). Syntenic groups were then further categorized into three subgroups: (1) syntenic and transcribed (ST), (2) syntenic and allelic-transcribed (SA), and (3) syntenic to a PCG (Fig. 1d, e). NATs and intronic RNAs were excluded from this analysis because of their partial overlap with PCGs. The sequence similarity of ST lncRNA (mean 85.71%) was much lower than that of mRNA (mean 96.60%) (Additional file 2: Figure S3). The SA category constituted the majority (71.67–86.00%) of syntenic lncRNA loci in all four comparisons (Fig. 1e). Importantly, the SA lncRNAs comprised over 80% (1269 out of 1517) of syntenic homologous lncRNA loci (Fig. 1e) in the comparison between GhA_T_ (a sub-genome of the allotetraploid (AADD)_1_ genome) and GhD_T_, based on data from identical genome and tissues. The above data further confirmed that expression of lncRNAs are predominantly species-specific.

### LncRNAs are reprogramed in synthetic interspecies F_1_

**Fig. 2:**
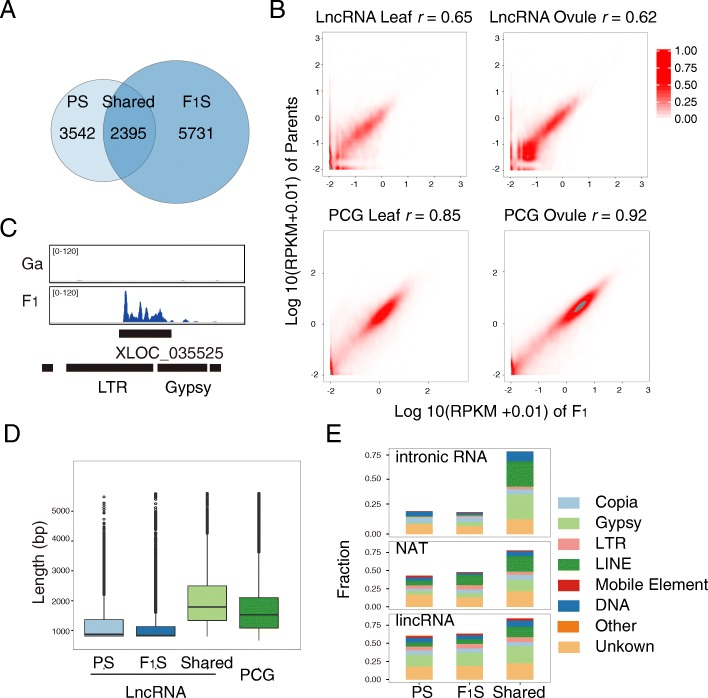
LncRNA reprogrammed in synthetic F_1._ **a** Venn diagram showing overlapped lncRNA assembled in parents and F_1._ LncRNAs were grouped into three catalogs. Parent-specific (PS), F_1_-specific (F_1_S), and shared lncRNA. **b** Dot plot representing the expression of lncRNA and PCGs in F_1_ compared to parents. *r* is the Spearman correlation. **c** Stacking diagram illustrating a TE-overlapped lncRNA allelic-transcribed in F_1_ hybrid. **d** Length distribution of parent-specific, F_1_-specific, and shared lncRNA in F_1_ and parents. PCGs are the control group. **e** Distribution of parent-specific, F_1_-specific, and shared lncRNA overlapped with transposons

Next, we asked whether the genome-specific patterns of lncRNAs were formed in the early stage of hybridization. A comparison of assembled lncRNAs between F_1_ and the parent genome was conducted. We identified in total 8514 lncRNA in F_1_; only 29.47% (*n* = 2,395, Fig. 2a) were overlapped with those of its parents. Furthermore, an in silico hybrid was constructed by mixing the diploid parental RNA-seq data in a ratio of 1:1, to reflect the accumulated gene expression divergence of the two parents without the impact of hybridization [52]. The lncRNA annotation files of Ga, Gr, and F_1_ were merged using cuffmerge [53]. In stark contrast to PCGs, we found lncRNAs exhibited more expression variance (*r* value by Spearman correlation, 0.62–0.65 for lncRNA versus 0.85–0.92 for mRNA) (Fig. 2b). For example, lncRNA XLOC_035525 was a novel transcript from the non-coding region of Ga stimulated in hybrid (Fig. 2c). Therefore, we observed a burst of lncRNA transcription in the interspecific hybridization of F_1_.

To exclude the possibility that the lncRNA burst was triggered by inbreeding, two near-isogenic lines (NILs) from inbred lines of cotton, upland cotton (Gh, accession Zhong12), and Zhong12 *GL* (a dominant, glandless line produced by multiple generations of backcrossing with Zhong12) were selected as control group. These NILs share identical genetic backgrounds. Using the same pipeline we had constructed earlier, a total of 4615 lncRNAs from the control group were identified (Additional file 1: Table S2). In the comparison between Zhong12 and Zhong12 *GL*, only 1.71% (73 out of 4281) of lncRNAs were differentially expressed (*p* < 0.05). In contrast to the lncRNA expression pattern in the NILs, the in silico parents and F_1_ exhibited a total of 34.75% (1999 out of 5752) differentially expressed lncRNA loci in leaves and 50.00% (2594 out of 5184) in ovules. The global changes in the lncRNAs of F_1_ suggest that interspecies hybridization stimulated a reprogramming of transcription on the non-coding region of the genome.

### The conserved lncRNAs are overlapped with TEs

To investigate the effective factors affecting lncRNA preservation in polyploidization, we anchored our analysis on the F_1_ genome. The shared lncRNAs in F_1_ and parents tended to have long transcripts and were overlapped with TEs (Fig. 2d, e). The proportions of F_1_-specific (F_1_S) and parent-specific (PS) lncRNAs overlapping with TEs were 63.49% and 60.41%, respectively, while in conserved lncRNAs, this proportion was as high as 84.47%. This phenomenon was also observed in intronic RNA and NATs (Fig. 2e). Considering all of these results, we hypothesized that TE was involved with lncRNA retention and burst in hybridization.

### LncRNAs are constrained on LINE and Gypsy-overlapped loci

Previous comparative studies of human, mouse, and zebrafish genomes indicated that non-TE lncRNAs might suffer relatively high evolutionary constraint than TE-derived lncRNAs do [40]. TE might contribute to the evolution of lncRNA in both the short (i.e., interspecies hybrid) and long term (i.e., polyploidization).

**Fig. 3:**
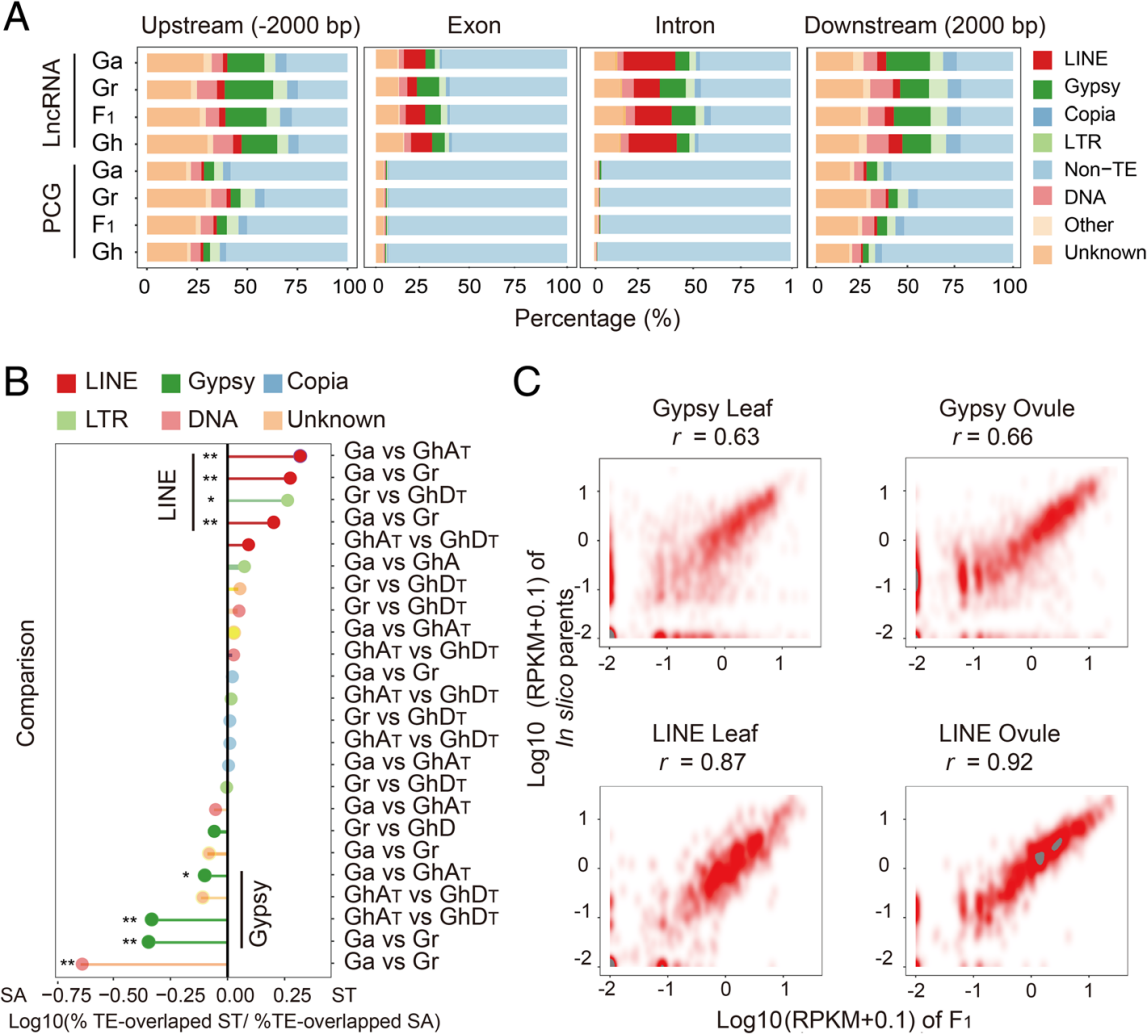
The impacts of LINE and Gypsy on lncRNA expressions in genome shock. **a** Distribution of transposons in different gene/lncRNA features. **b** The ratio of the proportion of TE-overlapped ST lncRNA and TE-overlapped SA lncRNA. Frequencies of many specific TE families in ST and SA lncRNA differ significantly (based on Fisher’s exact test). ST lncRNA are above 0 on the *X* axis, and SA lncRNA are below 0. **c** Dot plot representing the expression correlation of Gypsy- and LINE-overlapped lncRNAs in F_1_, compared to the parents. *r* is the Spearman correlation

TEs can be classified into two groups in general, DNA transposons and retrotransposons [47]. Retrotransposons can be further classified into LINE, SINE, and LTR according to structure variation [47]. We assessed the distribution of each type of TEs on lncRNA loci using PCGs as control. According to the analysis with BEDTools intersect [54], the lncRNA loci contained significantly more TE segments at the upstream 2000 bp regions, exon sequences, intron sequences, and downstream 2000 bp regions than did PCGs (Fig. 3a). This striking trend was in agreement with the reported observations of human, mouse, and zebrafish vertebrate genomes [40]. The Gypsy showed the largest proportion of lncRNA-overlapped TEs due to its largest share of TEs in the cotton genome (Fig. 3a). However, LINEs comprised ~ 40% of lncRNA-overlapped TEs on exons in each cotton species, although this type of TE only occupied 0.81–1.65% of the cotton genome as a whole (Fig. 3a). This indicated that LINE might distinctively impact the lncRNAs.

The association of TEs and lncRNA expression in the ST and SA groups were examined. The comparisons were conducted between the presence frequency of TEs in the ST and SA groups. The distribution of LINEs was skewed toward ST lncRNAs, while Gypsy was significantly enriched in the SA lncRNAs (Fisher’s exact test, typical *p* < 0.01) (Fig. 3b). The correlation coefficient of TE-overlapped lncRNA expression levels between the parents and F_1_ was calculated. Surprisingly, an even stronger correlation coefficient was observed for LINE-overlapped lncRNAs compared to Gypsy-overlapped lncRNAs, both in ovule (LINE *r* = 0.92; Gypsy *r* = 0.66) and leaf tissues (LINE *r* = 0.87; Gypsy *r* = 0.63) (Fig. 3c and Additional file 2: Figure S4). These association test results suggested that TEs in the categories of LINE and Gypsy represented distinct functional pattern in genome shock.

### LncRNAs are transcribed from siRNA-depleted LINEs/TEs

**Fig. 4:**
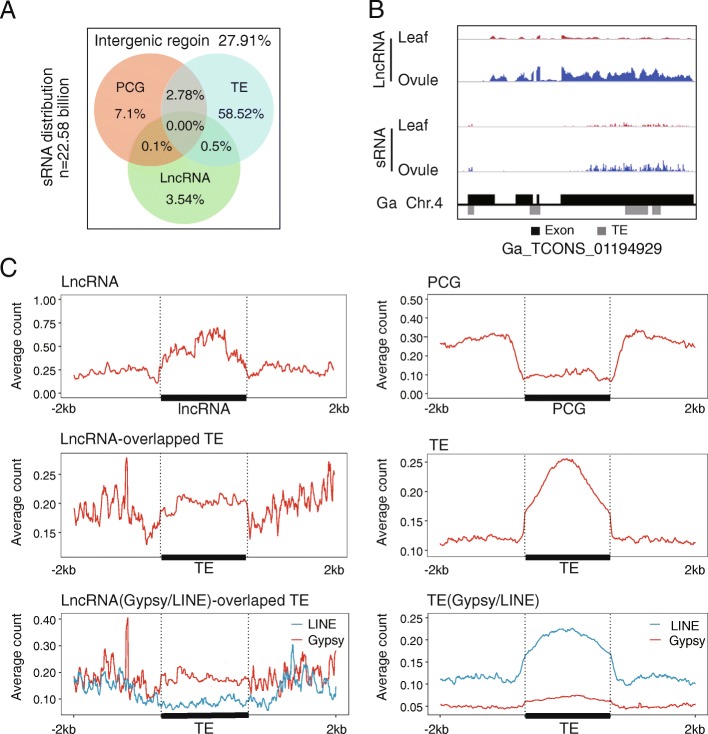
LncRNAs were transcribed from siRNA-depleted LINEs/TEs. **a** Venn diagram showing the sRNA distribution in PCG, lncRNA, and TE regions. **b** Stacking diagram illustrating a TE-overlapped lncRNA transcription and sRNA. **c** sRNA distribution pattern on lncRNA, mRNA, lncRNA-overlapped TE, and TE regions. Gypsy and LINE types of TE were selected in order to compare sRNA distribution on lncRNA-associated TE and genome-wide TE

TEs are generally considered to be major recruiters of epigenetic modifications, such as siRNA and DNA methylation [55–58]. We found that more TEs were overlapped with lncRNAs than PCGs (Fig. 3a). To define the relationship between epigenetic modifications on lncRNAs and TEs, we performed deep small-RNA sequencing for the leaf and ovule tissue of F_1_ (Additional file 1: Table S5). A total of 4.84 billion siRNA reads were obtained after filtering out tasiRNA, microRNA, and snoRNA (Additional file 1: Table S6). Most siRNAs were mapped to TE regions (on average 61.8%) (Fig. 4a), while PCGs and lncRNA-associated siRNAs occupied 9.89% and 4.14% of the total siRNAs respectively. As shown in Fig. 4b, lncRNA Ga_XLOC_435840 was a representative TE-overlapped lncRNA locus that generated siRNA covering both TE and non-TE regions (Fig. 4b).

Similar to findings in model plant genomes [59]), cotton siRNAs were abundantly enriched in TE bodies, but less in the upstream and downstream of TE bodies (Fig. 4c, Additional file 1: Figure S5). For the PCGs, sRNAs were enriched in the upstream and downstream regions of the gene body and less in the gene body (Fig. 4c, Additional file 1: Figure S5). Meanwhile, the siRNA distribution pattern on lncRNA loci was distinctive from that on both TEs and PCGs (Fig. 4c, Additional file 1: Figure S5). Compared to common TEs, lncRNA-associated TEs were covered by less siRNA (Fig. 4c, Additional file 1: Figure S5).

SiRNAs are known to suppress TE activity via siRNA-directed DNA methylation (RdDM) pathway in plant genomes. Accordingly, we predicted that the expression of TE-overlapped lncRNA could be affected by siRNA. Since LINE-overlapped lncRNAs were more stably transcribed compared to Gypsy-overlapped lncRNAs (Fig. 3c, Additional file 1: Figure S5), we hypothesized that siRNA distribution pattern on lncRNA-overlapped LINE and Gypsy might be different. To test this, we examined the distribution density of siRNA over the Gypsy and LINEs that overlapped with lncRNA regions respectively. As expected, the mapping densities of siRNAs in the transcribed regions of LINEs were much lower than those in Gypsy (Fig. 4c, Additional file 1: Figure S5). Twenty-four nucleotides and 21 nt siRNA were both enriched on lncRNA with similar pattern during the genomic shock (Additional file 1: Figures S6 and S7).

### LncRNAs were primarily transcribed from demethylated LINEs/TEs

**Fig. 5:**
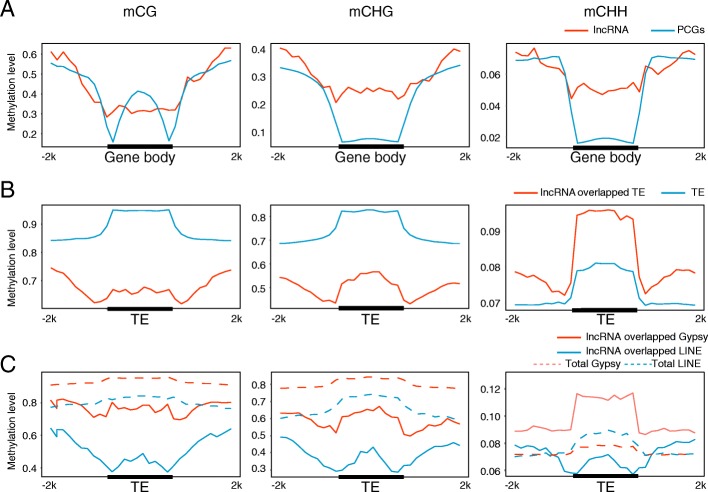
LncRNA were transcribed from demethylated TEs. Density plots of DNA methylation (CG, CHG, and CHH) profiles on lncRNA versus PCG (**a**), TE versus lncRNA-overlapped TE (**b**), and lncRNA-overlapped Gypsy versus lncRNA-overlapped LINE (**c**)

To validate the effects of siRNA on the activity of lncRNA and lncRNA-overlapped TE, we further compared the DNA methylation pattern on lncRNAs with PCGs and TEs in general using publicly available bisulfite sequencing (BS-seq) data of Ga, Gr, F_1_, and Gh [60]. CG methylation accounted for the majority of the 68,166 DMRs in hybridization (CG 63.66%, CHG 25.33%, CHH, 11.02%), which was consistent with reports of *Arabidopsis* and bean genomes [61, 62]. To dissect the DNA methylation dynamics in hybridization, we selected to show the pattern on lncRNAs and PCGs in F_1_. The CHG and CHH levels on the lncRNA body region were higher than that in PCGs (Fig. 5a). But for the lncRNA-overlapped TEs, the CG and CHG levels were much less on the TE body (Fig. 5b). Strikingly, the lncRNA-overlapped LINE showed low methylation level on all three contents (Fig. 5c). This trend was in line with the siRNA distribution patterns shown in Fig. 4c. Subsequently, we speculated the DNA methylation changes might impact the lncRNA activity in hybrid.

**Fig. 6:**
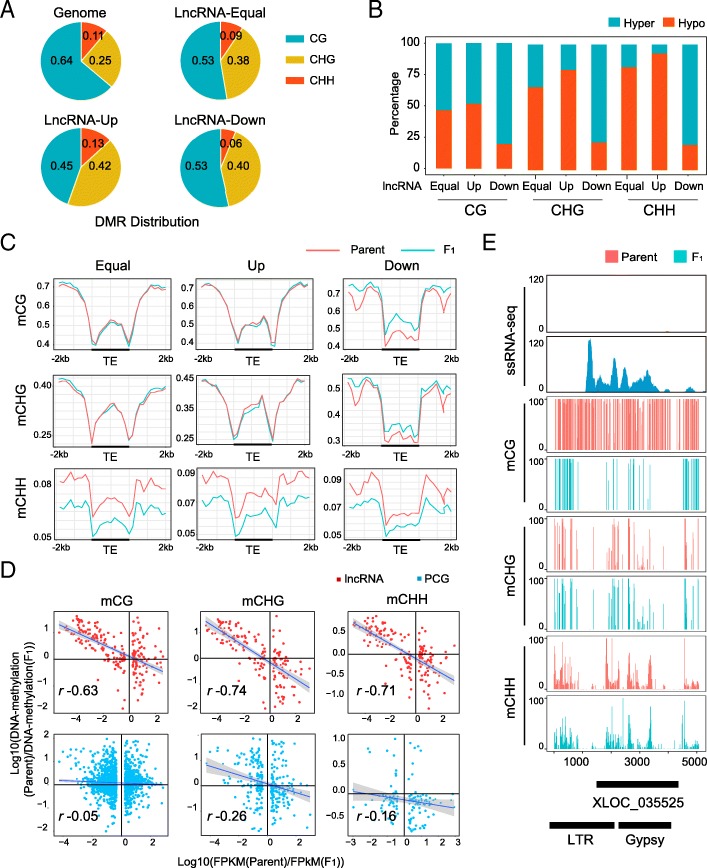
DNA methylation level is negatively associated with lncRNA expression in F_1_ hybrid. **a** Pie chart showing the distribution of differently methylated regions (DMRs) of lncRNAs in categories of CG, CHG, and CHH in F_1_ hybrid. LncRNAs were classified into groups of equal expressed (Equal, *n* = 2610), upregulated (Up, *n* = 1,302, *p* < 0.01, fold change > 2), and downregulated (Down, *n* = 1,272, *p* < 0.01, fold change < 0.5). **b** Histogram showing the proportion of hyper- and hypo-methylation on DMRs in lncRNAs. LncRNAs were classified into groups the same with **a**. **c** DNA methylation (CG, CHG) profile of equal expressed (Equal), upregulated (Up), and downregulated (Down) lncRNA in hybrid. Red and blue represent parent and F_1_, respectively. **d** Correlation between differential gene expression (*p* < 0.01 and fold change > 2.0) and differential DNA methylation (*p* < 0.01). **e** Example of F_1_-upregulated lncRNA, XLOC_035525. Stack view of XLOC_035525 locus with RNA seq and DNA methylation in Ga and F_1_

A dramatic DNA methylation change has been reported in the intraspecific hybrids of *A. thaliana* [62], rice [14], maize [13], and cotton [60], and CG methylation makes the greatest contribution to genome-wide DNA methylation changes in hybrids [13, 15, 60, 62]. In addition, Wang et al. [50] found that lncRNA could be induced by zebularine, a DNA methylation inhibitor, in cotton ovules. In light of this, we wanted to describe in detail the impact of certain methylation changes on lncRNA loci in hybridization. To address this question, we performed further association tests on the differentially methylated regions (DMRs) between the F_1_ hybrid and its parents (Ga and Gr). Conversely, the portion of DMRs arising from CG methylation was significantly lower on lncRNA loci (40%) in F_1_ compared to the total DMRs (64%). More specifically, the portion of DMR arising from CHH methylation increased from 11% of the total level to 22% on the F_1_ upregulated loci (Fig. 6a). Furthermore, we found that the DMR resulting from CHG and CHH in F_1_ lncRNA loci was predominantly hypo-methylated (Fig. 6b). These data implied that DNA demethylation was in fact active on activated lncRNA loci in the F_1_ hybrid genome.

To confirm this observation, we examined the DMR patterns on differentially expressed lncRNAs by plotting DNA methylation distributions. As shown in Fig. 6c, the lncRNA-overlapped TEs were significantly less methylated in the upregulated lncRNAs in F_1_, which was true in all three DNA methylation contents (Fig. 6c). The CHH methylation on lnRNAs was constantly low in the F_1_, which was consistent with the hypo-methylation status in general. This trend suggested the RdDM might be active on lncRNA genes in F_1_ hybrid.

### DNA methylation is negatively associated with lncRNA expression in F_1_ hybrid

Although genome-wide DMRs were identified in multiple hybridization tests, it is still inconclusive whether DNA methylation is associated with PCG expression changes in hybridization. We performed a correlation test for DNA methylation changes versus PCG and lncRNA expression changes in F_1_. There was no correlation between DNA CG methylation and the expression of PCGs in F_1_ (*r* = 0.03, *p* < 8.53 × 10^−02^) (Fig. 6d), but lncRNA was negatively correlated with DNA CG methylation (*r* = − 0.63, *p* < 2.2 × 10^−16^) (Fig. 6d). The correlation remained significant for CHG and CHH methylation (CHG *r* = − 0.74, *p* < 2.20 × 10^−16^; CHH for, *r* = − 0.71, *p* < 2.2 × 10^−16^). The representative example of XLOC_035525 activated in F_1_ clearly showed the difference in DNA methylation between the parent Ga and F_1_ (Fig. 6e). We therefore concluded that the DNA methylation level changes on lncRNA-overlapped TE regions were the major cause of lncRNA expression changes in F_1_. Specific demethylated TE regions contributed to the origin of novel lncRNA in the F_1_ genome.

### TE-derived lncRNAs as a source of functional genes

**Fig. 7:**
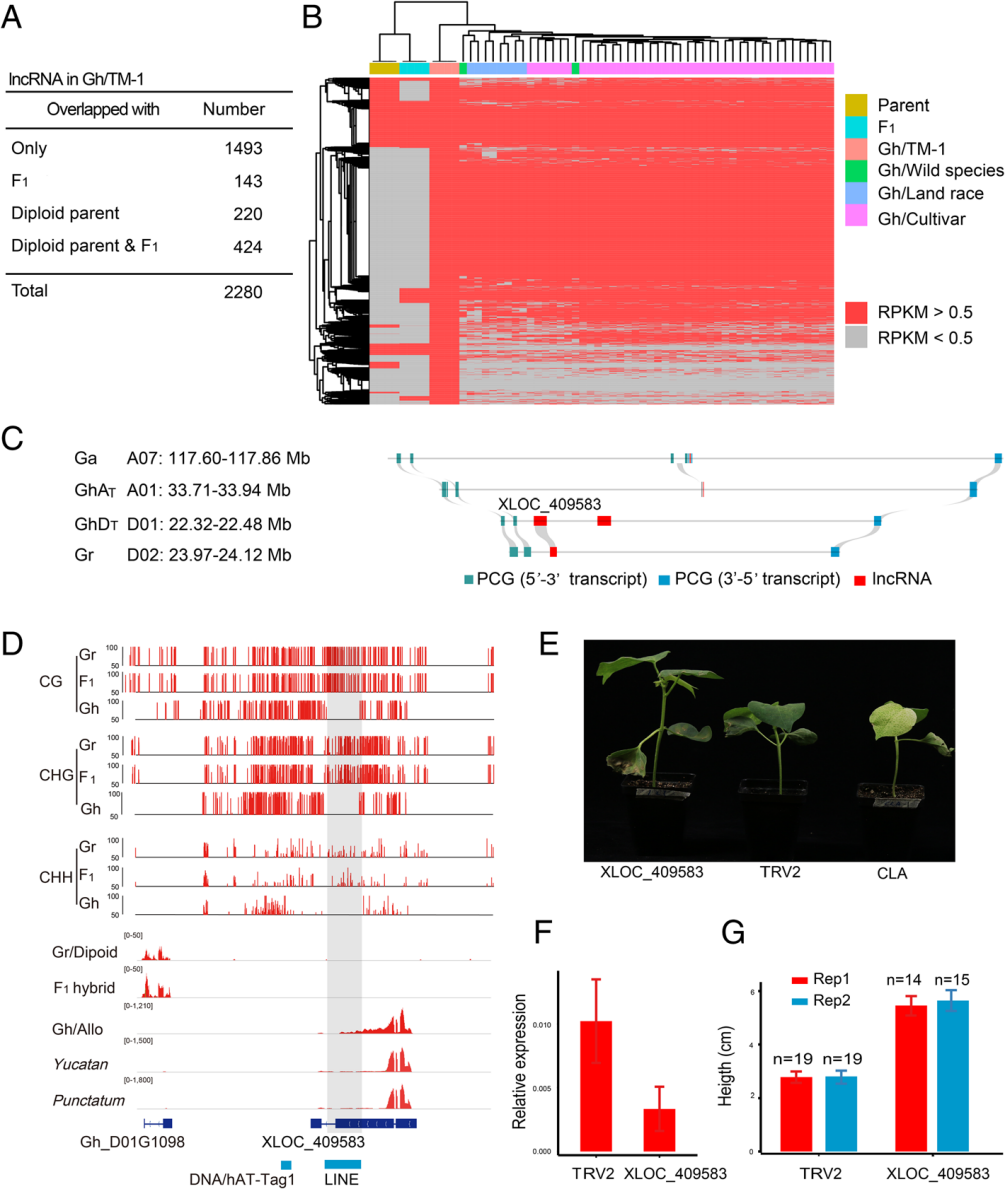
Polyploidization-stimulated lncRNAs have potential function. **a** Table showing number of Gh lncRNA overlapped with parent and F_1_. Total number of SA and ST lncRNA of Gh expressed in leaf (threshold RPKM > 0.5). **b** Heatmap showing lncRNA expressed in diploid parents, F1, Gh, upland cotton land races, and cultivars. **c** The syntenic relationship of XLOC_409583 in Gossypium spp. Gray curves connect the homologous regions between the corresponding chromosomes. The syntenic region was defined as the region with at least four aligned coding genes. XLOC_409583 is located on the D_T_ subgenome (on chromosome of GhD_T_ D01), which is syntenic with the D diploid ancestor (on Gr Chr02). **d** Browser stacks for DNA methylation and RNA-seq of XLOC_409583 in Gr (diploid ancestor), F_1_ hybrid, and TM-1 (allotetraploid). **e** Representative photo of TM-1 plants treated with virus-induced gene silencing (VIGS) constructs TRV2: XLOC409583, TRV2, and TRV2:CLrV. **f** Relative expression of XLOC409583 in VIGS-treated cotton group. Plants treated with an empty vector were used as a control group. **g** Histogram showing the height of XLOC_409583 in VIGS-treated cotton group. An empty vector was used as a control group. *n* represents the number of plants in each block

Since hybridization stimulated the transcription of non-coding regions of the genome, we ask whether these non-coding transcripts were simply noises arising from the chaos of genome shock or fixed in the tetraploid population as a source of functional genes. To address this question, we examined the lncRNA expression profile of allotetraploid cotton in wild species: 4 land races and 40 cultivars (Additional file 1: Table S7) [63]. The lncRNA homologs in the diploid parents and F_1_ were employed as a control. By comparing their expression activity in the putative diploid ancestors, we found that 1493 out of the 2280 lncRNAs (syntenic either with Ga or Gr, RPKM > 0.5) were specifically expressed in Gh (Fig. 7a). lncRNA expression was relatively stable in the allotetraploid, not only in the cultivars, but also in the wild cotton *yucatanense* and land races (Fig. 7b, Additional file 1: Table S8). But lncRNA expression varied drastically between the diploid and allotetraploid species (Fig. 7a, b, Additional file 1: Table S8). These results suggest that the genome shock of polyploidization introduced a significant variation in lncRNA expression similar to the effect of hybridization between the diploid species.

To further investigate whether the non-coding transcripts stimulated by genome shock have potential functions, we selected 10 lncRNAs (Additional file 1: Table S9) from the ST and SA groups in Gh vs F_1_ for functional tests and comparison. One lncRNA among these candidates, XLOC_409583, was expressed from a demethylated TE locus. The primary sequence of XLOC_409583 was identified in both the D_T_ and Gr (D) genomes, while the A_T_ subgenome lacked an apparent orthologous sequence (Fig. 7c). In the D_T_ subgenome, XLOC_409583 originated from a LINE locus (Fig. 7d). In contrast to F_1_ and its diploid ancestor Gr, the active expression of XLOC_409583 in the cultivated upland tetraploid cotton TM-1was associated with the demethylation of LINE. The active expression of XLOC_409583 was also detected in the wild upland cotton *yucatanense*, 4 land races (*latifolium, punctatum*, *morrilli*, *palmeri*), and 40 up-land cotton cultivars (Fig. 7d and Additional file 1: Table S8), indicating that XLOC_409583 transcription is stable after polyploidization.

To refine our understanding of the biological role of XLOC_409583, we performed virus-induced gene silencing (VIGS) tests in TM-1. The plants that underwent XLOC_409583 silencing showed increased height compared to the control group, indicating that the novel lncRNA XLOC_409583 played a role in plant development in the tetraploid cotton genome (*n* = 15 in each treatment, with two repetitions) (Fig. 7f, g). Discovery of the activation of XLOC_409583 by demethylation provides insight into the role of DNA demethylation in the emergence of novel lncRNA in hybrids and polyploids. Functional analysis of these novel lncRNAs will further uncover their biological significance in hybrids and polyploids [64].

### RNA polymerase II is essential for the transcription of TE-overlapped lncRNA

**Fig. 8:**
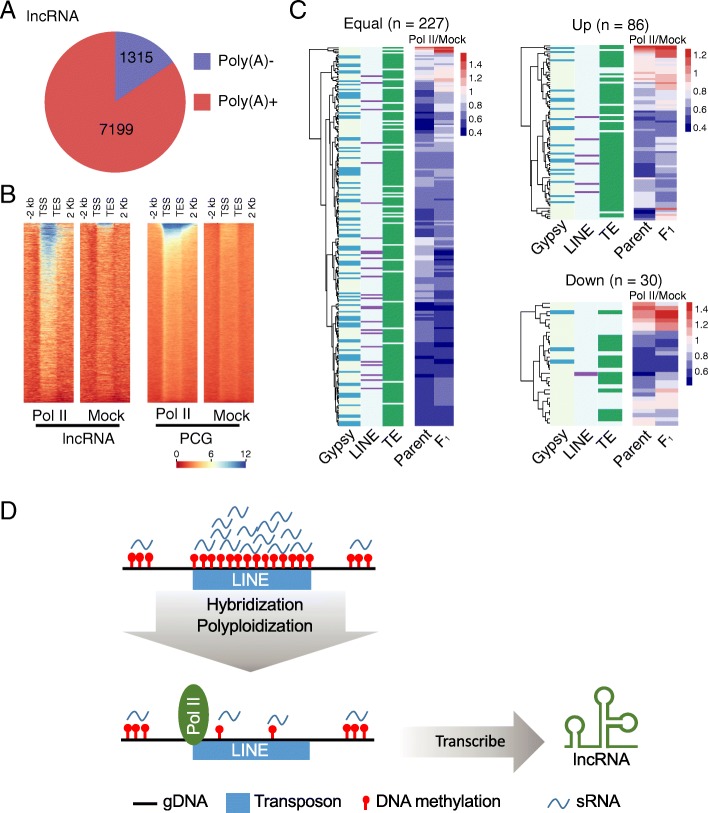
Pol II is essential for transcription of TE-overlapped lncRNA. **a** Venn diagrams illustrating the proportion of lncRNAs with reads mapped against mRNA Seq library prepared with poly(A) enrichment (poly(A)+). Poly(A)− represents the unmapped lncRNAs. **b** Enrichment of Pol II signals at lncRNA and PCG sites. The heatmap reads are clustered according to the enrichment profile of Pol II and mock. **c** Heatmap showing the dynamics of Pol II binding efficiency difference in equal and up- and downregulated lncRNAs in the F_1_ hybrid. The left panel of the heatmap indicates the annotation of lncRNA overlapped with Gypsy, LINE, and TE. **d** Schematic model of lncRNA origination within TE regions during genome shock of hybridization and polyploidization. **d** Transposon elements (TEs) are generally suppressed by siRNA and DNA methylation. During hybridization and polyploidization, de novo DNA methylation mediated by small RNA introduces dynamic RNA transcription. Based on our analysis, the reprogramming of DNA methylation during hybridization gives rise to novel lncRNAs from LINEs

To determine whether the variation in lncRNA expression seen in hybridization and polyploidization is affected by RNA transcriptase, we examined the lncRNA profile in the natural population of cotton. LncRNAs with mapping reads in the mRNA-Seq profiles were identified as Poly (A)+. Eighty-five cotton mRNA-seq were scanned in total [44, 63]. We found most lncRNA (84.55%) could be detected in the Poly (A)+ library (Fig. 8a), which was in agreement with previous reports for rice and maize [63].

To confirm that lncRNAs were transcribed by RNA polymerase II (Pol II), we used Pole II antibody to pull down the binding DNA fragments in diploid cotton (Ga and Gr), the F_1_, and the allotetraploid (Gh) cotton species (Additional file 1: Table S10). Then, using the model-based analysis for ChIP-Seq, we identified 1952–7576 high-confidence peaks (Additional file 1: Table S11). Pol II signals were enriched on both lncRNA and PCG in similar patterns (Fig. 8b, Additional file 2: Figure S8). Compared to the diploid parents, the binding efficiency of Pol II in F_1_ is not associated with transcription efficiency on either lncRNA loci or on PCGs (Pearson’s correlation test, *p* > 0.05). In addition, most Pol II-associated lncRNA transcripts contain TEs (Fig. 8c). These observations suggest that Pol II is the major RNA polymerase binding to lncRNA loci, especially on TEs (Fig. 8d). These results imply that, in addition to Pol IV and V, Pol II is also involved with TE transcription.

## Discussion

### Interspecies hybrid is a model for the study of lncRNA evolution

Evolutionary conservation of lncRNA is poorly understood due to the lack of sufficiently close species with finely sequenced genomes for study [33]. Taking advantage of the three published genomes of closely related cotton species [44–46], we identified that 83.97% of cotton lncRNAs were conserved in *Gossypium* spp. Furthermore, approximately 59.29 to 76.34% of PCGs were one-to-one syntenic in all four genomes. These highly syntenic genomes helped to identify the homologous lncRNA loci.

Using the collinear method, homologous lncRNA loci with low sequence similarities were identified at a high confidence level. Our research model applied to the F_1_ and allotetraploid genomes was designed to facilitate the examination of homologous lncRNA loci. Therefore, the genome specificity observed in this system provided solid evidence of the fast turnover of lncRNA. We found that only 10.86–26.15% of syntenic lncRNA loci were constantly expressed in multiple cotton species. These results further confirm the previous report that in animal genomes both sequence divergence and expression turnover contribute to the species specificity of lncRNA [32].

### Rapid turnover of lncRNA in hybrid

Hybridization and polyploidization are both common and crucial in genome evolution. Genome-wide changes can be ascribed to variations in PCG expression and alteration of epigenetic modifications, such as DNA methylation, histone modification, and sRNA generation. Most PCGs in synthetic allopolyploid are expressed at mid-parent level [5]. However, in this study, we found that lncRNA expression was changed dramatically in hybrid. Our data indicated that lncRNA was not substantially gained and lost during evolution, but was instead induced by the genomic shock of interspecies hybridization, provoking new species formation. Transcription of lncRNA underwent tremendous variation during genome shock. Given that lncRNAs participate in critical biological process, such as *Xist* silencing in animals [65] or miRNA target mimicry in plants [26], it is reasonable to assume that lncRNA reprograming in hybrids can affect genes regulated by non-coding RNAs. Therefore, we hypothesized that the rapid transcriptional turnover of lncRNAs might further affect the lineage-specific emergence or disappearance of specific traits.

### The epigenetic modifications on TE affect the lncRNA origin

TEs have been reported to be involved in miRNA origin and evolution [66, 67]. TEs also contribute to alternative gene structures such as novel promoters, splice sites, or polyadenylation signals [68]. Previous reports elucidated that TEs are major contributors to the origin of some lncRNA in vertebrates [38–40]. Many functional lncRNAs such as *Xist* [41], *TUG1* [69], *linc-ROR* [70], *PCAT-1* [24], and *SLC7A2-IT1A* [71] are overlapped with TEs. We also observed a strong correlation between TEs and lncRNAs along the evolutionary path from diploid to allotetraploid. In our simulation model of cotton evolution, lncRNAs tended to be retained with TEs, indicating the potential impact of TEs on lncRNA origin as well as heritability. We found that TEs exhibited biased distribution toward lncRNA loci rather than coding genes, and LINEs especially contributed disproportionally to lncRNAs in all cotton species.

As a mobile element, TEs are normally transcriptionally silent regions due to DNA methylation via RdDM. But TEs overlapping with lncRNA loci are transferred to a transcriptionally active status, implying a possible difference in local regulation or modification. The sRNA distribution pattern and DNA methylation levels of lncRNA loci in the F_1_ hybrid confirmed that these regions were activated. Therefore, we hypothesized that the lncRNA loci originated from select TEs, such as LINEs, with few suppressive modifications. Based on our analysis, the de novo methylation as well as reprogramming of DNA methylation in hybridization created novel lncRNAs arising from LINEs (Fig. 8). A latest study on the epigenetic landscape of cancer cells finds that the lncRNA genes are hypo-methylated [25]. Some oncogenic lncRNA genes are under the diverse epigenetic modifications, such as CpG methylation. The de-methylated lcnRNA gene *EPIC1* can promote the cell propagation in cancer [25]. These reports suggested that the DNA methylation-directed lncRNA regulation is a general mechanism both in plant and animal genomes.

## Materials and methods

### Materials

Interspecies hybrids of *Gossypium arboreum* (AA, 2n = 2x = 26) and *G. raimondii* (DD, 2n = 2x = 26) were generated by hand pollination. Three biological replicates of 0 DPA (days post anthesis) ovules and leaves from each of *G. arboreum*, *G. raimondii*, interspecies hybrid (*G. arboreum* × *G. raimondii*) F_1_, and *G. hirsutum* (AADD, 2n = 4x = 52) were collected from the greenhouse of Nanjing Agricultural University. All plants were under the same controlled growing conditions at 25 °C, 16/8 h day/night. Samples were frozen in liquid nitrogen immediately upon collection and stored at − 70 °C in preparation for RNA isolation.

### LncRNA library construction and sequencing

Total RNA was isolated from the plant tissues using the Spectrum Plant Total RNA Kit (Sigma-Aldrich). After RNA isolation, ribosomal RNA was removed using the Epicentre Ribo-zero™ rRNA Removal Kit (Epicentre, USA). Next, sequencing libraries were generated from rRNA-depleted RNA using the NEBNext® Ultra™ Directional RNA Library Prep Kit for Illumina® (NEB, USA). Finally, strand-specific sequencing was performed with the Illumina HiSeq 2000 system (paired-end 125-bp reads).

### Pipeline for lncRNA identification

All strand-specific RNA-seq reads were quality-trimmed and quality-filtered using fastx_toolkit (http://hannonlab.cshl.edu/fastx_toolkit/). Clean reads were then mapped separately to the corresponding references using TopHat (v.2.0.14) (*N* = 2, library type = fr-first strand). The genome sequences and annotation files for *G. arboreum* (v.2.0), *G. raimondii*, and *G. hirsutum* (v.1.1) were collected from the Cotton Genome Project (CGP: https://www.ncbi.nlm.nih.gov/genome/?term=Gossypium+arboreum), Phytozome (v.9.0) (http://www.phytozome.net), and the website (http://mascotton.njau.edu.cn/Data.htm), respectively. The F_1_ genome was generated by mixing the genome sequences of *G. arboretum* and *G. raimondii*. The transcripts from each dataset were assembled independently using the Cufflinks program (v.2.2.1) (-u, library type= fr-first strand) and Scripture program (--coverage =0.2) [53, 72]. All transcripts from each species were merged to generate final transcripts using Cuffmerge [53]. Transcripts less than 200 nt were discarded. Using Cuffcompare, transcripts were given the class code “u,” “x,” or “i,” representing intergenic sequences, antisense sequences of known genes, and intronic sequences, respectively [53]. The Coding Potential Calculator (CPC) software was used to calculate the coding potential of the remaining transcripts [73]. All transcripts with CPC scores > 0 were discarded. The remaining transcripts were subjected to HMMER (v. 3.1b2) in order to exclude transcripts containing known protein domains (cutoff < 0.001) [74]. The remaining transcripts were candidate lncRNAs. To reduce the isoform complexity of lncRNA, only the longest transcript of each loci was used for further analysis.

### Identification of transposable element-derived lncRNA

We annotated transposable elements in the genome using RepeatMasker (v.4.0.6) (http://www.repeatmasker.org). RepeatModeler (v.1.0.8) (http://www.repeatmasker.org/RepeatModeler.html) was used to create three de novo transposable element (TE) libraries based on the *G. raimondii*, *G. arboreum*, and *G. hirsutum* reference genomes using default parameters. We then used RepeatMasker to identify repeat elements using both the de novo libraries and the MIPS repeat database (mipsREdat_9.3p) [75]. The annotation from RepeatMasker was then parsed to exclude low complexity and non-TE repeats. Next, transposons were classified into Gypsy, Copia, LTR, LINE, DNA, unknown, and other categories. LncRNA-derived TEs were identified by determining overlapping genomic coordinates of TEs or TE fragments of at least 1 bp using the intersectBed program from BEDTools (v.2.17.0) [54]. When multiple TE features were found for a single lncRNA, the longer TE feature was counted.

### Identification of ST and SA lncRNAs

ST lncRNA was reconstructed based on sequence similarity and position. NATs and intronic RNAs that overlapped protein-coding gene (PCG) loci were removed. Then the PCGs and lincRNAs in different subgenomes were reciprocally aligned using BLASTN (v.2.2.27) (*E* value < 1 × 10^−10^, -max_target_seq 1) [76]. ST lncRNA, in syntenic blocks between two subgenomes, were identified using the MCScanX (-b 2, -s 5) [77]. To identify SA lncRNA, the lncRNA sequences of species A were aligned to the syntenic blocks of species B using BLASTN (*E* value < 1 × 10^−10^, -max_target_seq 1) [76].

### Expression analysis

HTSeq-count software (v.0.6.0) [78] was used to obtain read counts for each lncRNA or gene module (-s yes –m union). Read counts were normalized to RPKM (reads per kilobase per million reads). To assess the accumulated gene expression divergence between the parent lines and the hybrid F_1_, an in silico parental mix was constructed by combining clean reads of *G. raimondii* and *G .arboreum* at a ratio of 1:1. Spearman’s correlation between biological replicates was calculated using *R* from the RPKM values. Differentially expressed transcripts were calculated using the R package, edgeR [79].

### Small RNA library construction and sequencing

Total RNA was extracted from the 0 DPA ovules and leaves of two biological replicates. Small RNAs were then separated from total RNA by polyacrylamide gel electrophoresis. Three micrograms of total RNA per sample was used as the input material for construction of the small RNA library. Sequencing libraries were generated using NEBNext® Multiplex Small RNA Library Prep Set for Illumina® (NEB, USA), following the manufacturer’s recommendations. The library preparations were sequenced on an Illumina HiSeq 2000 platform and 50-bp single-end reads were generated.

### Processing of sRNA sequencing data

After sRNA sequencing, adapters and low-quality nucleotides were trimmed from the data. sRNA clean reads were then aligned with the F_1_ genome (a mixture of the Ga and Gr genomes) using Bowtie, with no mismatch (-m 50, -v 0) [80]. Any aligned small RNA reads that mapped to more than 50 loci were removed. The remaining mapped reads were aligned with noncoding RNAs using Rfam release (http://rfam.sanger.ac.uk/) and the known miRNA database in miRBase release 19 (http://www.mirbase.org/) [81], in order to identity miRNA, snRNA, tRNA, and rRNA. miREvo [82] and mirdeep2 software [83] were integrated to predict novel miRNAs. All reads originating from miRNA, TAS genes, rRNA, tRNA, snRNA, and snoRNA were removed. The remaining 20–25-nt-long reads were selected as siRNA. The distribution of siRNA across different features was drawn using deeptools [84].

### Analysis of MethylC-seq reads

MethylC-seq and differentially methylated regions (DMRs) were retrieved from a previous study in the supplemental information (https://genomebiology.biomedcentral.com/articles/10.1186/s13059-017-1229-8) [60]. The methylation level across different features was calculated using an in-house perl and R script.

### Virus-induced gene silencing technology

A 300-bp fragment of XLOC409583 was amplified (F primer, AATAAG TGTGAAATTGTCGGGC; R primer, ATTCATGGCGATAAAGTCGGA) and cloned to form a *Xba*I/*Bam*HI-digested pTRV2 vector, creating a VIGS vector named *pTRV2- XLOC409583* (F primer: ATTCTGTGAGTAAGGTTACCGAATTCGAAA GTCCTTCGCTACAAAT; R primer: AGACGCGTGAGCTCGGTACCGGATCC ACTATTGCCAATCGTCTTCA). The vectors *pTRV1* and *pTRV2-XLOC409583* were then transformed by the *Agrobacterium* strain GV3101 via electroporation (Bio-Rad, Hercules, CA, USA) [85]. For the VIGS assay, the transformed *Agrobacterium* colonies were incubated overnight at 28 °C in an antibiotic selection medium containing 50 mg/L rifampicin and 50 mg/L kanamycin. *Agrobacterium* cells were centrifuged and resuspended in infiltration buffer (10 mM MgCl_2_, 10 mM MES, and 200 mM acetosyringone), adjusted to an OD_600_ = 0.5. *Agrobacterium* strains containing *pTRV1* and *pTRV2* vectors were mixed in a ratio of 1:1. Seedlings with mature cotyledons but without a visible true leaf (7 days after germination) were infiltrated by inserting the *Agrobacterium* suspension into the cotyledons via syringe. The plants were grown in pots at 25 °C in a growth chamber under a 16/8 h light/dark cycle with 60% humidity. For each treatment group, 32 individual plants were employed.

### RNA extraction and qRT-PCR

RNA was extracted from leaf tissue and treated with a BioFlux kit. First-strand cDNA was generated using TransScript One-Step gDNA Removal and cDNA Synthesis SuperMix (TransGen Biotec Co., Ltd.), according to the manufacturer’s instructions. Quantitative RT-PCRs were performed with the primers F: CCTTGTCAGAGTCCTCTGGTAG; R: GAGTTGAATGGGCATTCTTG.

### Chromatin immunoprecipitation and sequencing (ChIP-Seq)

Chromatin immunoprecipitation (ChIP) was performed as described and with several adaptations [86]. One gram of leaves for each sample of AA, DD, AD, and AADD genomes was used in the assay. After plant material crosslinking, nuclei isolation, cell lysis, and chromatin sonication as described in the protocol, ChIP reaction was performed using Anti-RNA polymerase II antibody (ABcam, Anti-RNA polymerase II CTD repeat YSPTSPS antibody [8WG16] - ChIP Grade, ab817) and protein A+G magnetic beads (Millpore), referred to as the “ChIP” group. The control group for each sample was set up similarly with the experimental group using sonicated chromatin with protein A+G magnetic beads but without antibody, referred to as the “Mock” group, which served as the background of the ChIP reaction. The ChIP reaction was performed overnight at 4° with gentle rotation, followed by separation and washing of beads using magnetic separation device (Millpore Magna GrIP Rack). DNA purification was performed using a commercial spin column kit. To verify the DNA enrichment, ChIP-Seq libraries were constructed with the NEBNext ChIP-Seq Library Prep Master Mix Set for Illumina (NEB) using NEBNext Multiplex Oligos for Illumina (NEB). DNA libraries including the “ChIP” and “Mock” groups respectively for each sample were pair-end sequenced with 150 bp reads using an Illumina HiSeq2500.

### Analysis of ChIP-Seq

All ChIP-Seq reads were quality-trimmed and quality-filtered using fastx_toolkit (http://hannonlab.csh.edu/fastx_toolkit/). Clean reads were then mapped separately to the corresponding references using Bowtie (v.2.0.14) with no mismatch [80]. Peak calling analysis was used for model-based analysis for ChIP-seq (MACS) [87]. Profile of ChIP-Seq in PCG and lncRNA was visualized using Deeptools with default parameter [84].

### Scan lncRNA expression variation in upland cotton population

One wild upland cotton (accession *yucatanense*),4 land races (*latifolium*, *punctatum*, *morrilli*, *palmeri*) and 40 up-land cotton cultivars were download from SRA project SRX2326742 [63] (Additional file 1: Table S7). Reads were quality-trimmed and quality-filtered using fastx_toolkit (http://hannonlab.cshl.edu/fastx_toolkit/). Clean reads were then mapped to the corresponding references of *Gossypium hirsutum* using TopHat (v.2.0.14) [53]. HTSeq-count software (v.0.6.0) [78] was used to obtain read counts for each lncRNA or gene module (-s yes –m union), then read counts were normalized to RPKM. Expressed lncRNA were determined by applying a threshold of RPKM > 0.5.

## Additional files

Additional file 1: **Table S1**. The strand-specific RNA sequencing (ssRNA-seq) library data summary. **Table S2**. The total lncRNA predicted in *Gossypium* species. **Table S3**. The total lncRNA predicted in *Gossypium* species. **Table S4**. Summary of the syntenic blocks of *Gossypium* spp. **Table S5**. The small RNA sequencing library data summary. **Table S6**. Classification of sRNA. **Table S7**. Eighty-five Poly(A)+ RNA-seq library of *Gossypium hirsutum*. **Table S8**. FPKM of Gh syntenic lncRNA. **Table S9**. Candidate lncRNAs. **Table S10**. Summary of ChIP-Seq library. (XLSX 1135 kb)

Additional file 2: **Figure S1**. LncRNA landscape in *Gossypium* spp., *G. arboreum*, *G. raimondii*, (*G. arboreum* × *G. raimondii*) F1, and *G. hirsutum*. **Figure S2**. Sequence similarity of lncRNAs across different species. **Figure S3**. Low similarity of syntenic and transcribed lncRNA. **Figure S4**. Expression correlation coefficient of different TE-overlapped lncRNA. **Figure S5**. LncRNA were transcribed from siRNA-depleted LINEs/TEs. **Figure S6**. Profile of 21 and 24 nt leaf sRNA. **Figure S7**. Profile of 21 and 24 nt ovule sRNA. **Figure S8**. Heatmaps showing the dynamics of Pol II binding signals in lncRNA and PCG features across the four cotton species. (PDF 2882 kb)
