# Supplementary material for: LncRNAs in polyploid cotton interspecific hybrids are derived from transposon neofunctionalization
Source: Genome Biol. 2018 Nov 12;19:195. doi: 10.1186/s13059-018-1574-2 (PMC6233382; doi:10.1186/s13059-018-1574-2)
Supplement: Supplementary file 2 — Figure S1. LncRNA landscape in Gossypium spp., G. arboreum, G. raimondii, (G. arboreum × G. raimondii) F1, and G. hirsutum. Figure S2. Sequence similarity of lncRNAs across different species. Figure S3. Low similarity of syntenic and transcribed lncRNA. Figure S4. Expression correlation coefficient of different TE-overlapped lncRNA. Figure S5. LncRNA were transcribed from siRNA-depleted LINEs/TEs. Figure S6. Profile of 21 and 24 nt leaf sRNA. Figure S7. Profile of 21 and 24 nt ovule sRNA. Figure S8. Heatmaps showing the dynamics of Pol II binding signals in lncRNA and PCG features across the four cotton species. (PDF 2882 kb) [file 13059_2018_1574_MOESM2_ESM.pdf]

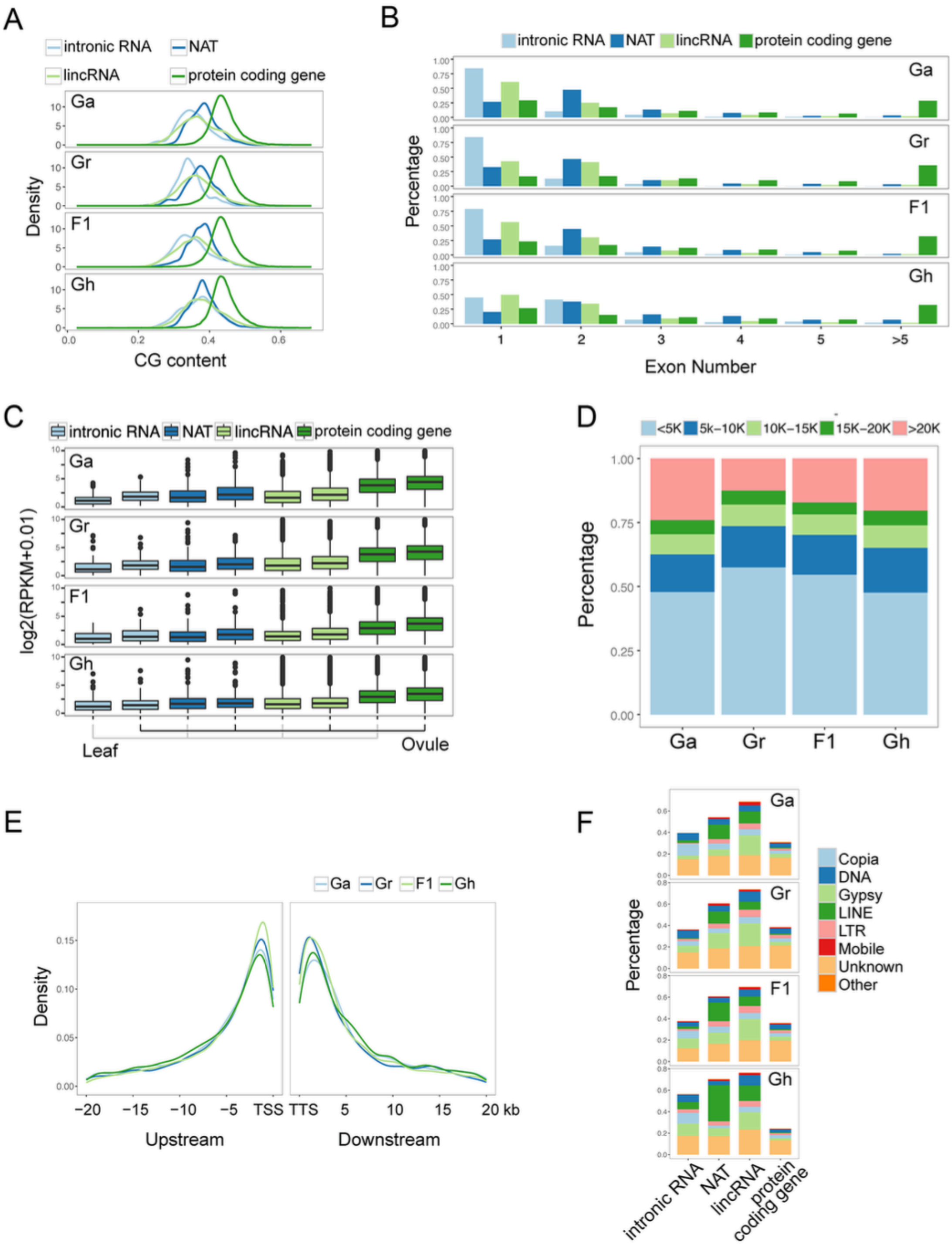

Fig S1: lncRNA landscape in *Gossypium* spp, *G. arboreum*, *G. raimondii*, (*G. arboreum* x *G. raimondii*)F$_1$ and *G. hirsutum*. (A): Line plot of the cytosine (C) and guanine (G) content in intronic RNA, NAT and lincRNA. The protein coding genes (PCGs) is used as control. (B): Bar chart comparing the numbers of exons in the intronic RNA, lincRNA, NAT and PCGs from four cotton species. (C): Box plot of expression levels of all intronic RNA, NAT, lincRNA and PCGs and natural antisense

transcripts (NAT) across the selected cotton species. (D): The distance distribution percentage to the flank PCGs of lncRNAs in the selected cotton species. (E): Density plots of the lincRNA by the distance to the flank PCGs across the four cotton species. TSS, translational starting site. TTS, translational terminal site. (F): The histogram comparing the relative coverage with transposon element (TE) of lncRNA and PCGs in four cotton species. The color codes indicate the TE categories.

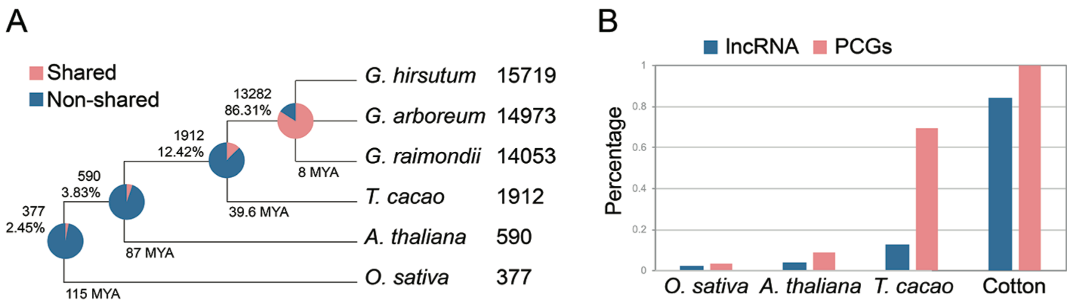

Fig S2: Sequence similarity of lncRNAs across different species. (A) Simplified phylogenetic tree. Internal branches, root and pie chart of the percentage of orthologous lncRNAs in each minimum comparison. Tree tips, lncRNA numbers 1-1 in compared species. The evolutionary age between species is shown under the branch. MYA, million years ago. (B) Exonic sequence conservation (similarity percentage), for lncRNA loci and protein coding gene in cotton, *T. caca*, *Arabidopsis thaliana* and rice.

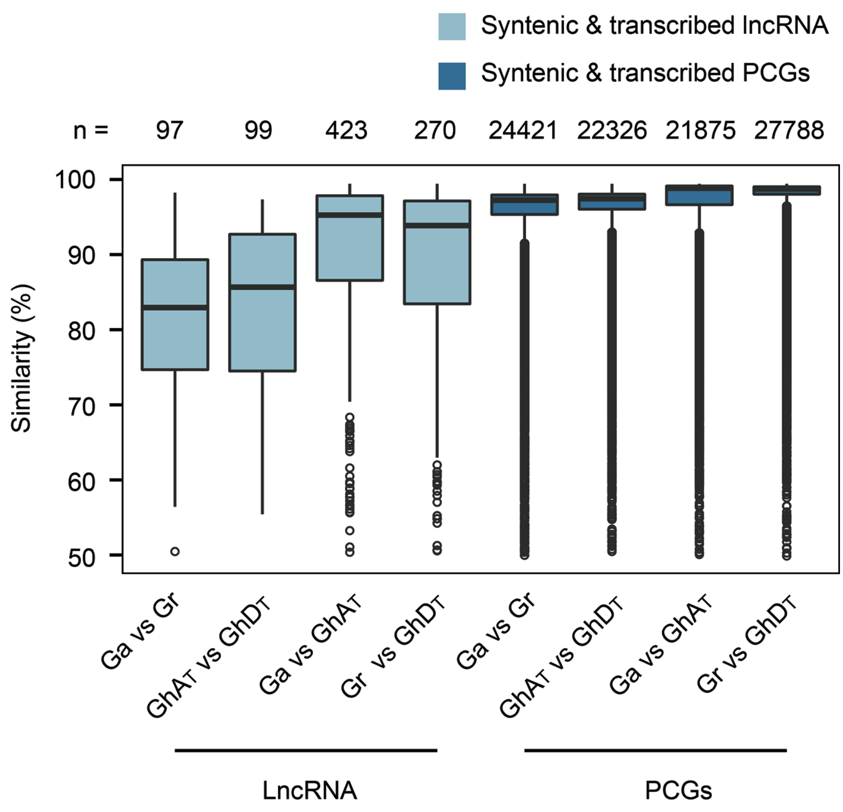

Fig S3: Low similarity of syntenic & transcribed lncRNA. Box plot of the sequence similarity of the lncRNAs and protein coding genes in the corresponding genomic comparisons.

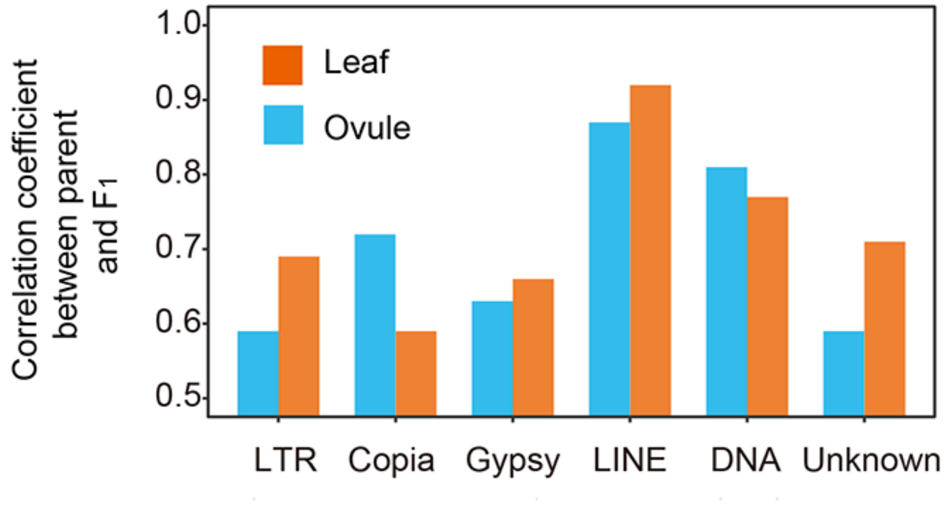

Fig S4: Expression correlation coefficient of different TE-overlapped lncRNA.

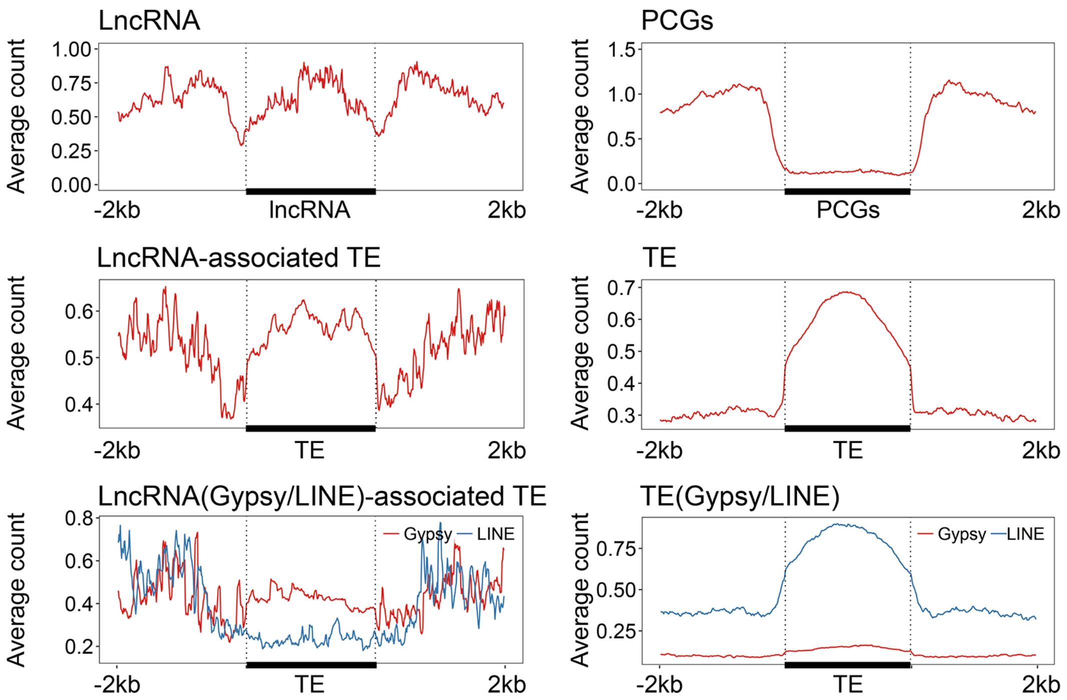

Fig S5: LncRNA were transcribed from siRNA-depleted LINEs/TEs. Ovule sRNA distribution pattern on lncRNA, PCGs, lncRNA- overlapped TE, and TE regions. Gypsy and LINE types of TE were selected in order to compare sRNA distribution on lncRNA-overlapped TE and genome-wide TE.

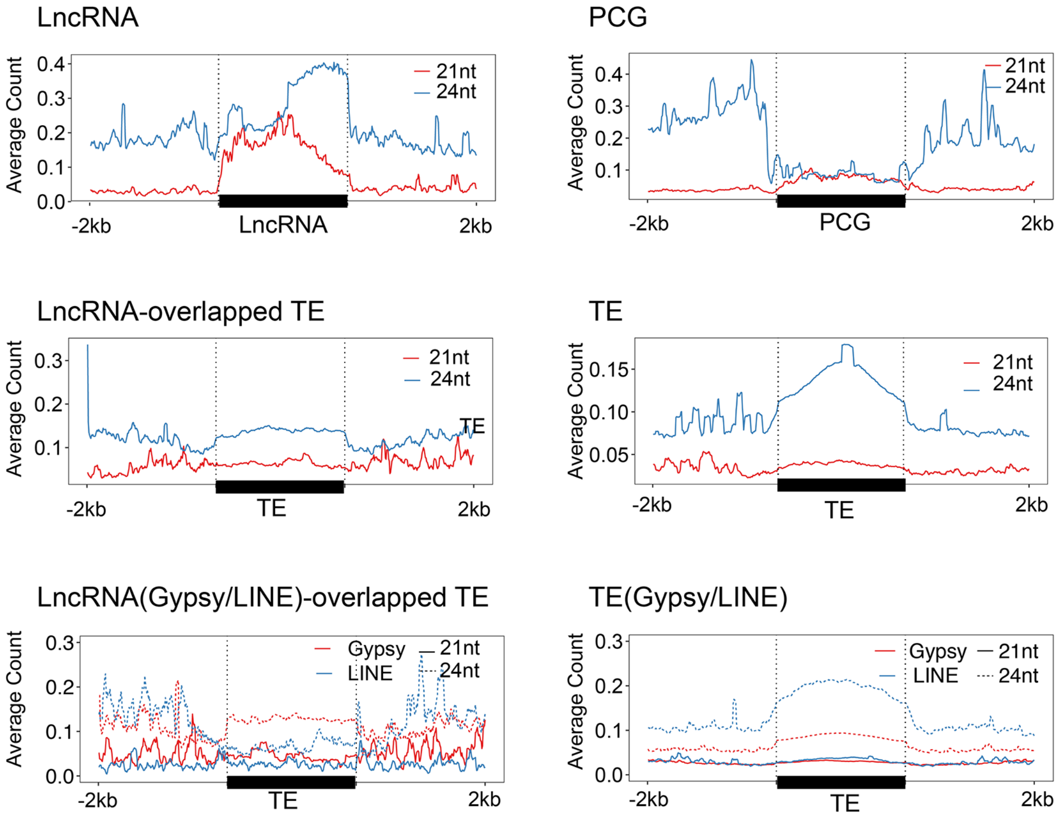

Fig S6: Profile of 21 and 24 nt leaf siRNA. 21 and 24 nt leaf sRNA distribution pattern on lncRNA, PCGs, lncRNA-overlapped TE, and TE regions. Gypsy and LINE types of TE were selected in order to compare sRNA distribution on lncRNA-overlapped TE and TE in genome wide.

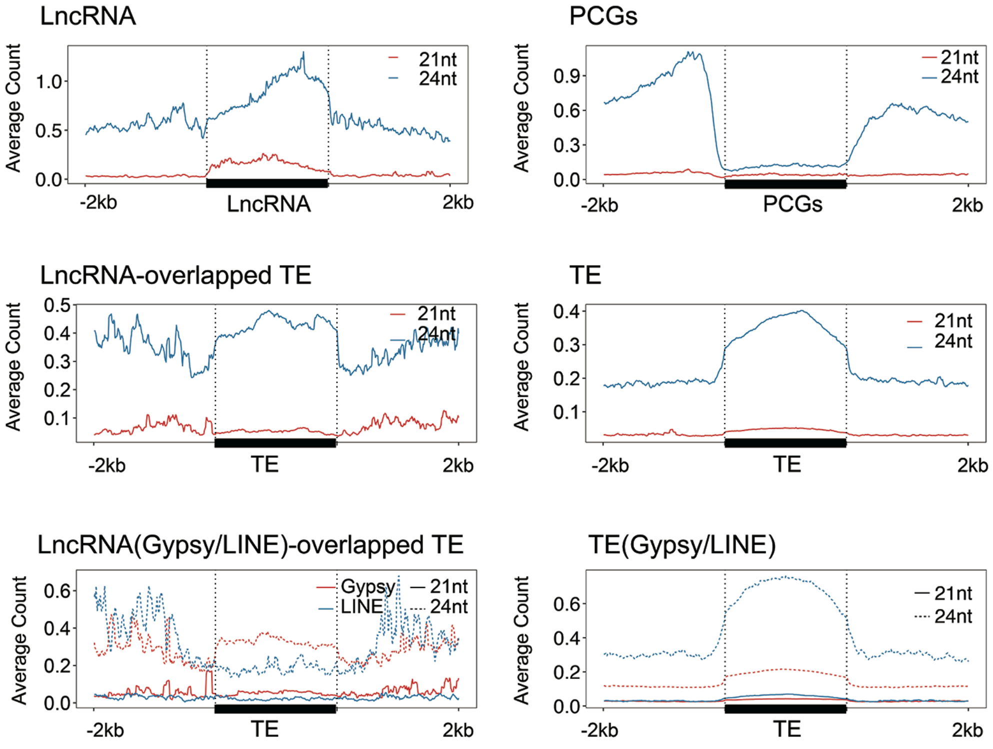

Fig S7: Profile of 21 and 24 nt ovule sRNA. 21 and 24 nt ovule sRNA distribution patterns on lncRNA, PCGs, lncRNA-overlapped TE, and TE regions. Gypsy and LINE types of TE were selected in order to compare sRNA distribution on lncRNA-overlapped TE and genome-wide TE.

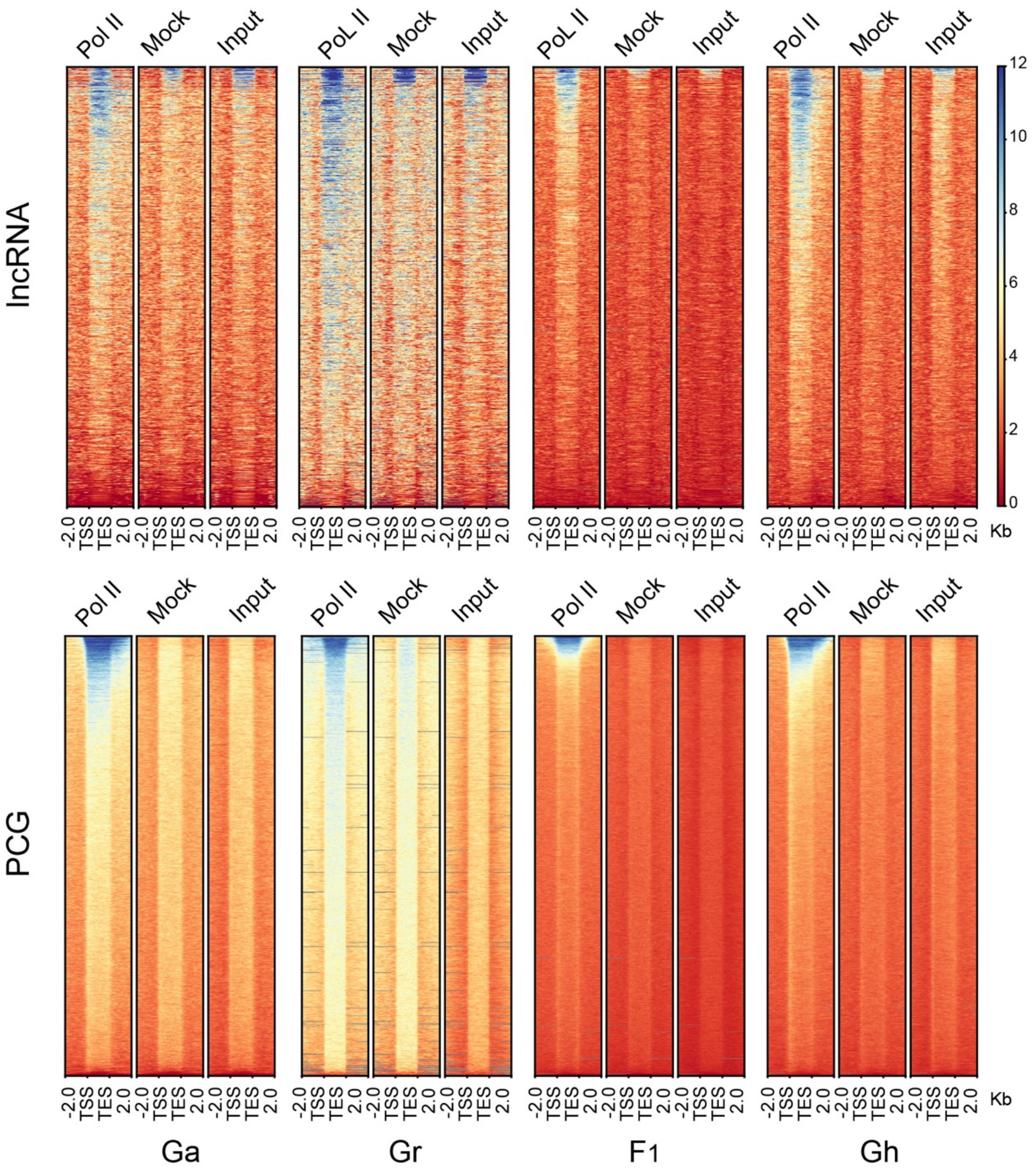

Fig S8: Heatmaps showing the dynamics of Pol II binding signals in lncRNA and PCG features across the four cotton species.
